# Health Literacy Scale and Causal Model of Childhood Overweight

**Published:** 2017-01-28

**Authors:** Ungsinun Intarakamhang, Patrawut Intarakamhang

**Affiliations:** ^1^ Behavioral Science Research Institute, Srinakharinwirot University, Bangkok, Thailand; ^2^ Department of Physical Medicine and Rehabilitation, Phramongkutklao College of Medicine and Hospital, Bangkok, Thailand

**Keywords:** Health literacy, Health behavior, Childhood obesity, Overweight children, Health scale

## Abstract

**Background:** WHO focuses on developing health literacy (HL) referring to cognitive and social skills. Our objectives were to develop a scale for evaluating the HL level of Thai childhood overweight, and develop a path model of health behavior (HB) for preventing obesity .

**Study design:** A cross-sectional study.

**Methods:** This research used a mixed method. Overall, 2,000 school students were aged 9 to 14 yr collected by stratified random sampling from all parts of Thailand in 2014. Data were analyzed by CFA, LISREL .

**Results:** Reliability of HL and HB scale ranged 0.62 to 0.82 and factor loading ranged 0.33 to 0.80, the subjects had low level of HL (60.0%) and fair level of HB (58.4%), and the path model of HB, could be influenced by HL from three paths. Path 1 started from the health knowledge and understanding that directly influenced the eating behavior (effect sized - β was 0.13, P<0.05. Path 2 the health knowledge and understanding that influenced managing their health conditions, media literacy, and making appropriate health-related decision β=0.07, 0.98, and 0.05, respectively. Path 3 the accessing the information and services that influenced communicating for added skills, media literacy, and making appropriate health-related decision β=0.63, 0.93, 0.98, and 0.05. Finally, basic level of HL measured from health knowledge and understanding and accessing the information and services that influenced HB through interactive, and critical level β= 0.76, 0.97, and 0.55, respectively.

**Conclusions:** HL Scale for Thai childhood overweight should be implemented as a screening tool developing HL by the public policy for health promotion

## Introduction

Obese children are‏ a global health problem that‏ carries‏ complications, especially in type 2 diabetes^1^‏. There were rapid increasing numbers of obesity prevalence among aged‏ 6‏-19 yr‏ since 1976‏-1980‏. Later, in 2003‏-2004, the percentage of obese children aged‏ 6‏-11 yr from 6‏.5‏% to 18‏.8‏%, and aged‏ 12‏-19 yr from 17‏.4‏% to 33‏%^2^‏. There were more than 155 million overweight children globally‏. In Europe,‏ where the obesity rate in children had increased by 400,000 annually and female children increased‏ weight more than male^3^‏. In Asia, 8911 children, aged 2‏-6‏ yr from 30 cities‏ of Iran in 2012‏-2013 showed the prevalence of obesity as‏ 5‏.7‏% and 5‏.2‏%,‏ respectively^4^‏.

The key factors of childhood overweight were low social and economic status, lack of parental nutritional awareness, and role in promoting healthy eating among children‏ in school^5^‏. In Thailand, obesity prevalence was lower than Europe, South and North America yet similar to Japan and Korea but higher than Sri Lanka^6^‏. Obesity at the age over 6 yr old would become obese adults at the rate of 25‏%^7^‏. While obesity at the age of 12, would turn out to be obese adults more than 75% and both of them would be at high risk of developing type 2 diabetes,‏ coronary heart disease, and hypertension‏. Moreover, obese people were more‏ likely to suffer from pathogenic infection and other complications implied that obesity would become a burden to developing countries‏’ GDPs by more than 1‏.1‏% to 1‏.2‏%, without any appropriate measures, the issue could refer to‏ stop the potential halt the economy of these developing countries^7^‏.

Health literacy (HL) among overweight children and teenagers, therefore, is the key for understanding in the preventive behaviors‏. According‏ to research on HL, for the first time in the proceedings of a health education conference^8^‏, WHO defined HL‏ as the cognitive and social skills that determine the motivation and ability of individuals to gain access, understand and use information in promoting‏ good health^9^‏. Thailand added HL as the realization of individual‏’s knowledge, skills, as well as confidence in practicing beneficial behavior^1^‏. HL is the individual‏’s social performance and critical analysis skills determining the degree to which people are able to access, evaluate and communicate information in order to promote health^11^‏. Moreover, HL as the skills needed for searching, assessing and integrating health information as well as the desire to understand specific health and cultural terminologies within a particular health system^12^‏. Health outcome is related to health knowledge that is crucial to HL formation^13^‏. HL might develop over time by which people could manage their health, access and keep track of information and services, communicate with health professionals and receive a proper treatment, as well as exchange information among members or make social interaction^14^‏.

HL‏ within this‏ research framework considers‏ the six skills include‏ cognitive,‏ access,‏ communication,‏ decision,‏ self‏-management,‏ and media literacy skill^14-17^‏. The model of Nutbeam^11^‏ consists of three levels‏: functional HL, interactive HL, and critical HL‏. This interactive HL was shown‏ in 90 obese women‏ in Iran via a‏ family‏-centered model of interactive educational classes and the dietary behavior scores of the intervention group had significant improvement^18^‏. Therefore, the aims‏ of this study were to develop an HL scale for Thai childhood overweight, evaluate the HL level and develop the path model of HL‏ for obesity preventive behaviors‏.

## Methods

The research used‏ mixed methods including the process of synthesizing related documents and researches, the analysis of the factors and causal relationship model‏. Overall,‏ 2,000 school students were selected from all parts of Thailand and collected in 2014‏. This could be divided into 3 phases‏.

Phase 1: Knowledge was synthesized from theories, documents, and related research for developing an‏ HL‏ scale for overweight children along with a qualitative research method with the following details‏. To synthesize the HL‏ measurement tools for overweight children by systematically review of‏ literature starting from theories‏ and researches related HL‏. The used literature‏ were published in a full‏-text on PubMed and Science Direct from 2000‏-2013‏. Six papers aimed to develop an‏ HL scale were‏ Rapid Estimate of Adolescent Literacy in Medicine (REALMTeen)^19^, eHealth Literacy Scale (eHEALS)^20^, the Chinese version (short form) of the Test of Functional HL in Adolescent (CS‏-TOFHLAd)^21^,‏ test of Functional Health Literacy in Adults (S‏-TOFHLA)^22^, Health Literacy Scale for Overweight 9^th^ Grade Thai Students^16^‏ and the ABCDE‏-Health Literacy Scale for Thais, the recommendations for Thai people at risk of obesity and hypertension^23^‏. These‏ papers‏ were‏ analyzed to obtain HL index for overweight children‏. The synthesized‏ measurement tools‏ were‏ assessed by five‏ Thai experts in HL and analyzed to obtain a more consistent version‏. Public hearings were held by related professionals to discuss the updated measurement tools‏.

Phase 2: Drafting and development‏ of‏ a complete version of HL tool for overweight children‏. The details for this phase were as follows‏: An‏ HL tool was drafted by a focus group and assessed by experts in the field of HL tool for children‏’s health, behavior, and psychology, and was developed for the completed version of HL tool‏. The completed version of HL tool was‏ trialed with the 100 samples of overweight children to test for reliability of the questionnaire‏.

Phase 3:‏ Consistency between the path analysis model and the structural equation model of preventive behaviors of obesity were checked through the modification of questions‏. The sample size was determined based on the size required to confirm a causal relationship model^24^, an adequate sample size to‏-parameters‏ ratio would be 20:1‏.‏ There were‏ 25 parameters;‏ therefore, the participants should be 500 per group‏. The questionnaires‏ were collected from 2000 samples, which‏ were‏ children of ages 9 to 4 yr with BMI of 23‏-25 kg‏/m^2^obtained by quota‏-stratified random sampling‏. These children were currently studying in schools‏ subject‏ of different sectors in different areas from all parts of Thailand as follows‏: 1‏) Office of the Basic Education Commission, 500 samples, 2‏) local government, 500 samples, 3‏) Office of the Higher Education Commission, 500 samples, and 4‏) Office of the‏ Vocational Education Commission, 500 samples‏. All samples were collected from both urban and provincial areas.‏ The questionnaires were done in 2,000 students‏ from‏ 40‏ schools (50 students in each school)‏ in 10‏ provinces of 5 parts; north, south, east, west, and central of Thailand‏.

The ethical consideration for this research was approved by the Institutional Review Board‏ of Srinakharinwirot University with the declaration of Helsinki regarding ethical principles for research in human‏. In addition, the written informed consent was obtained from all the participants prior to the study‏.

The analysis of primary data involved descriptive statistics such as frequency distribution, percentage, mean, and standard derivation‏. Confirmatory factor analysis (CFA) and structural equation model were also used‏. The calculation was done by Linear Structural Relationship Model ‏(LISREL‏). Statistical values included‏ the absolute fit index Chi‏-Square (χ^2^) Goodness of Fit Index: GFI‏≥0‏.90, Root Mean Squared Error Approximation: RMSEA‏≤ 0‏.05, [Incremental fit index] Comparative Fit Index‏: CFI‏≥0‏.90, [or Parsimony fit‏ index] Parsimony Normed Fit Index: PNFI‏≥0‏.50, and adjusted goodness of Fit Index‏: AGFI‏ ≥0‏.90, and χ^2^‏/*df*‏ ≤ 5^24^‏.

## Results

The index of consistency (IOC)‏ was 0‏.80‏-1‏.00 of all items‏. For the reliability testing of trialed tools‏ showed that 65 questions were selected with the item‏-total correlation‏ coefficient = 0.2-0.8‏ and‏ Cronbach‏’s alpha coefficient = 0.70 up. In addition,‏ the result of‏ the tested‏ instrument for constructs validity by the second CFA‏ technique‏. The‏ assumptions‏ of a‏ CFA‏ included multivariate normality and a sufficient sample size by KMO‏-Kaiser Meyer Olkin‏=0‏.92 and significant Bartlett‏’s testing of Sphericity (*P*<0‏.05). A‏ scree plot‏ showed the decreasing‏ rate which variance of three principal components accounts for over 21‏.79‏% of the explained variance‏. Therefore, the samples were from populations with equal variances and adequate sample size‏. HL tool for children of ages 9‏-14 yr‏ was consistent and acceptable level‏ as follows: 10 questions about knowledge related the obesity prevention‏ which were binary questions‏ with multiple choices (true‏ choice‏=1 and false choice‏=0), with discrimination index (r=0‏.45‏-0‏.80), factor loading‏ =0‏.39‏-0‏.66, reliability of KR‏-20‏=0‏.76. Five questions about accessing the information and services‏ that influenced the preventive behaviors of obesity, with‏ r‏=0‏.40‏-0‏.57, factor loading‏ =0‏.66‏-0‏.73, reliability of Cronbach‏’s Alpha (α=0‏.74). Six questions about‏ interaction‏ skills that the communicating for added skills, with r‏=0‏.50‏-0‏.60, factor loading‏ =0‏.61‏-0‏.80, α‏=0‏.79. Five questions about managing their health conditions, with‏ r‏ =0‏.52‏-0‏.64, factor loading‏=0‏.70‏-0‏.78, α‏=0‏.79. Five questions about media literacy for the prevention of obesity, with‏ r‏=0‏.53‏-0‏.64, factor loading‏=0‏.66‏-0‏.73, α‏=0‏.82. Four questions about making appropriate health‏-related decision, with‏ r‏=0‏.26‏-0‏.36, factor loading‏ =0‏.42‏-0‏.50, α‏=0‏.70. Twenty questions about preventive‏ behaviors of obesity, with r‏=0‏.30‏-0‏.51, factor loading‏ =0‏.42‏-0‏.71, α‏=0‏.71‏-0‏.87 (Table1‏ and 2).

**Table1:** The qualitative measurement of the health literacy and obesity preventive‏ behaviors

**Component of health literacy and obesity preventive behaviors**‏	**Correlation** **Coefficient (r)**	**Factor loading** **of Item**
1‏.** Health knowledge and understanding (KR-20 = 0.76)**
1.1 Which disease may be associated with childhood obesity?‏	0‏.80	0‏.39
1.2‏ How to eat safely for‏ healthily to lose weight?	0.68	0‏.45
1‏.3 Which diet menu is safe and effective‏ for weight control?	0‏.63	0‏.66
1‏.4 Which are the foods causing obesity?	0‏.57	0‏.60
1‏.5‏ Which is the safe exercise in obese‏ children?	0‏.63	0‏.56
1‏.6 How to do the best exercise in obese children?	0‏.47	0‏.39
1‏.7 For warm up, when to do either before or after exercise?	0‏.60	0‏.63
1‏.8 Who is the best for emotional management?‏	0‏.45	0‏.41
1‏.9 Which is the best practice to relieve stress?	0‏.53	0‏.45
1‏.10 Which type of foods should be avoided in obese children?	0‏.75	0‏.51
2. Accessing the information and services (Cronbach‏’s Alpha‏ = 0‏.74)
2‏.1 Whenever you need to access the health information, how often could you access the health resource?	0‏.48	0‏.73
2‏.2 Whenever you need to know about the health information, how often could you ask an expert?	0‏.54	0‏.66
2‏.3 Whenever you have‏ the problem about searching health information, how often could you ask the‏ teacher or others?	0‏.40	0‏.67
2‏.4 How often do you reassure the health information from several sources?	0‏.57	0‏.72
2‏.5 How often do you review the health information or products before you make decision to believe and‏/or buy?	0‏.49	0‏.66
3‏. Communicating for added skills (Cronbach‏’s Alpha‏ = 0‏.79)
3‏.1 Whenever you‏ get an advice about obesity, how often could not‏ you understand?	0.50	0.61
3‏.2 How often do you ask the teacher or others for help about food label reading, calories calculating or health care?‏	0.54	0.80
3‏.3 How often do you translate knowledge of obesity and weight control for your family or friend to understand?‏	0.59	0.74
3‏.4 Whenever you read‏ the health brochures about obesity protection, how often could not you understand?	0.52	0.69
3‏.5 How often do you communicate about practice in obesity to your friends or others to understand?‏	0.60	0.77
‏ 3‏.6 How often do you inspire your friend with obesity to accept practices for healthy weight control?‏	0.58	0.76
4‏. Managing their health conditions (Cronbach‏’s Alpha‏ = 0‏.79)
4‏.1 How often do you consider the appropriateness of the nutritional value on foods?	0‏.54	0‏.73
4‏.2 How often do you set exercise goals and achieve?‏	0‏.55	0‏.72
4‏.3 How often do you manage‏ your stress appropriately?	0‏.52	0‏.70
4‏.4 How often do you review your practices for obesity protection and better health?	0‏.64	0‏.76
4‏.5 How often do you improve your environment for healthy?	0‏.60	0‏.78
5‏. Media literacy (Cronbach‏’s Alpha = 0.82)
5‏.1 How often have you seen an advertisement on the TV and search for information from the multiple sources to verify‏ prior to believe?	0‏.64	0‏.70
5‏.2 How often have you seen an advertisement to seek additional information for credibility before you buy?	0‏.63	0‏.68
5‏.3 How often have you logically analyzed the media information before making decision in‏ practice?‏	0‏.62	0‏.73
5‏.4 How often have you participated for health, you approve the activities prior to believe and practice?	0‏.53	0‏.66
5‏.5 How often have you discussed and criticized an advertisement for health before making decision in‏ practice?	0‏.60	0‏.73
6‏. Making appropriate health‏-related decision (Cronbach‏’s Alpha‏ = 0‏.70)
6‏.1 When do you go to a party at neighbor‏’s home and get an invitation to eat unhealthy foods such as, too sweet or high fat, how do you make decision?‏	0‏.32	0‏.50
6‏.2‏ When do your friends invite you to eat birthday cake, how do you make‏ decision?‏	0‏.32	0‏.45
6‏.3‏ When do your friends like to drink the soft drinks, how do you advise?‏	0‏.36	0‏.42
6‏.4 If you have the stress with weight gain, how should you choose to act?	0‏.26	0‏.43
7‏. Eating behavior (Cronbach‏’s Alpha = 0.87)
7‏.1‏ Eating fatty, fried foods and coconut milk‏.	0‏.38	0‏.52
7‏.2 Fish sauce, sugar added to food before eating‏.	0‏.33	0‏.52
7‏.3 Eating fresh fruits and vegetables every day, at least 500 grams per day‏.	0‏.33	0‏.44
7‏.4 Calories food controlling for proper body functioning	0‏.33	0‏.42
7‏.5 Drinking soft drinks, honeydew, chocolate, sweet milk	0‏.38	0‏.63
7‏.6 Eating fast foods, Pisa, hamburger, hotdog etc	0‏.45	0‏.69
7‏.7‏ Eating sweet, candy, ice‏-cream etc	0‏.47	0‏.65
7‏.8 Eating sweet bread, donut, cookies etc	0‏.51	0‏.62
7‏.9‏ Eating snack, fried potatoes‏.	0‏.41	0‏.49
7‏.10‏ Monitoring to overeat and overweight by yourself‏.	0‏.42	0‏.61
7‏.11 Eating fast in all meals‏.	0‏.44	0‏.53
7‏.12 Eating by concerning the useful food‏.	0‏.43	0‏.64
7‏.13‏ Eating the cooked, steam, baked, grill, boil food	0‏.44	0‏.47
7‏.14‏ Eating a snack or foods before bed‏.	0‏.40	0‏.52
8‏. Exercise behavior (Cronbach‏’s Alpha = 0.71)
8‏.1‏ Physical activity‏ and exercise continually to feel tried and sweaty‏.	0‏.30	0‏.50
8‏.2‏ Exercise continually to feel tried with a frequency‏ at least 5 d‏ per week‏ and 30 min a day	0‏.31	0‏.50
8‏.3 Playing sport, exercise after school‏.	0‏.37	0‏.57
8‏.4‏ Walking up the stair instead of lifting up, walking instead of riding‏.	0‏.31	0‏.53
9‏. Emotional coping (Cronbach‏’s Alpha‏ = 0‏.82)
9‏.1‏ Releasing emotion through eating‏.	0‏.42	0‏.71
9‏.2 Self‏- problem solving by optimization	0‏.33	0‏.50

**Table 2:** The 3 score levels for Thai childhood overweight for evaluating the health literacy level

**Total score**	**Meaning**
**Functional or Basic Level 1** ^st^ ** to2** ^nd^ **compound (Total score of 35 pts)**
<21 pts or <60‏% of the total score	Low level of cognitive skill
21 to 27‏.99‏ pts or‏≥‏ 60 to‏ <80‏% of the total score	Fair level of cognitive skill
28 to 35‏ pts‏ or‏ ≥ 80‏% of the total score	High level of cognitive skill
**Interactive Level 3rd to 4th compound (Total score of 55 pts)**
<33‏ pts or <60‏% of the total score	Low level of socially interactive and communicative skill
33 to 43‏.99 pts or ‏≥60‏% to <80‏% of the total score	Fair level of socially interactive and communicative skill
44 ‏– 54‏.9ptsor‏≥ 80‏% of the total score	High level of socially interactive and communicative skill
**Critical level 5th to 6th compound (Total score of 45 pts)**
<27pts or <60‏% of the total score	Low level of critical thinking skill
27 to‏ 35‏.99pts or ‏≥60‏% to <80‏% of the total score	Fair level of critical thinking skill
36 to‏ 45‏ pts‏ or‏ ≥80‏% of the total score	High level of critical thinking skill

**Figure 1:**
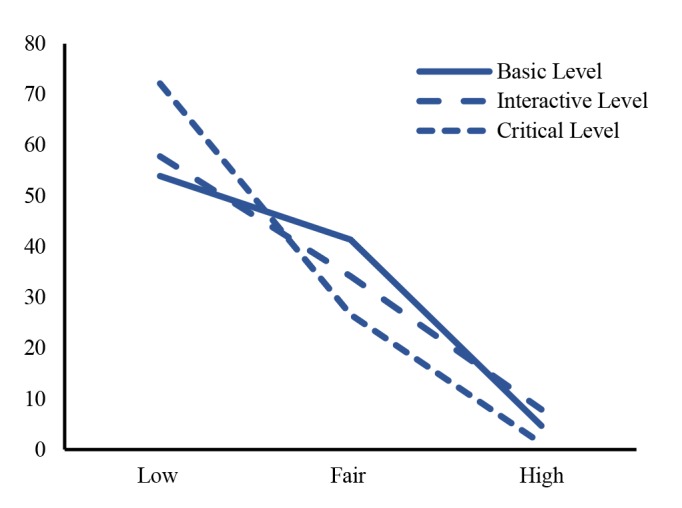
The health literacy‏ level of Thai childhood overweight

The assessment of HL for the prevention of obesity in children ages of 9 -14 showed that most of the subjects had low level of HL (60.4%), followed by those with fair level of HL, 38.3%, and those with high level of HL, only 1.3%. Most of them also had fair level of HB for preventing obesity, at 58.4%, while those with poor health behavior were at 39% and followed by those with good HB, at 2.6%. When considering about Basic‏ HL, most of the subjects were found to be on the low level more than 53.9%. Similarly, most of the subjects had low interactive and critical HL at 57.8% and 72.2% respectively. Still, 26.6% of the total samples had fair level of critical HL while only 1.2% had perfect critical health literate in Figure 1‏.

**Figure 2:**
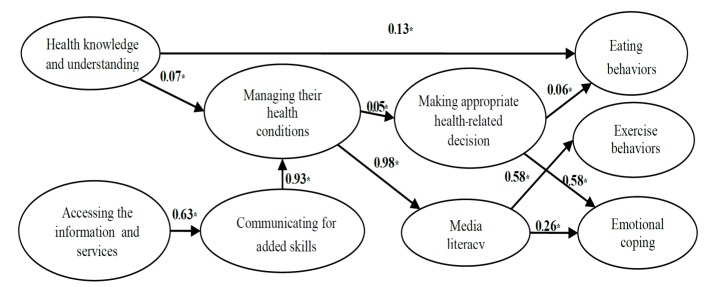
The path model of the composition of health literacy‏ affects the obesity preventive behaviors‏ of Thai childhood overweight‏ (*P< 0‏.05‏)

The analysis of the path model of the composition of HL affected the preventive behaviors of obesity. The hypothetical causal model was consistent with the empirical data based on parsimony fit index by χ^2^=60.10, P=0.00, df=12, RMSEA=0.05, CFI=0.99, AGFI=0.99, PNFI=0.72 and χ^2^/df =5. The development of the obesity preventive behaviors can be influenced by the deeper details of HL from three paths. Path 1 starts from the health knowledge and understanding that directly influences the eating behavior (β=0.13). Path 2 starts from the health knowledge and understanding that influenced managing their health conditions, media literacy, and making appropriate health-related decision (β=0.07, 0.98, and 0.05, respectively). Path 3 starts from accessing the information and services that influenced communicating for added skills, media literacy, and making appropriate health-related decision (β = 0.63, 0.93, 0.98, and 0.05, respectively) as follow in Figure 2.

**Figure 3:**
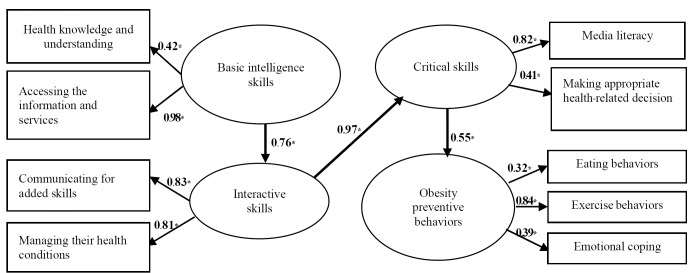
A causal relationship model for obesity preventive‏ behaviors of‏ Thai childhood overweight‏ (*P< ‏.05‏)

Three factors impact on HB: Functional level of HL measured from the health knowledge and understanding and accessing the information and services. Interactive level of HL and critical level of HL that influenced HB for preventing childhood obesity (β=0.76, 0.97, and 0.55, respectively) (Figure 3).

## Discussion

In this research, HL‏ for the preventive behaviors of obesity had been developed from Nutbeam‏’s concept of the 6 skills which are cognitive skill, access skill, communication skill, decision skill,‏ self‏-management skill, and media literacy skill^12^‏. Moreover, the study of the relationship between HL for the preventive behaviors of obesity, eating and exercising habits in children with over‏-nutrition by Thipwong and Numphol^25^‏ as well as the development of HL tool for junior high school students,‏ measured‏ by 5 components: (accessibility,‏ understanding,‏ assessment,‏ practicing, and communication)^26^‏.

For the assessment tool and factor extraction, the consistency and reliability were acceptable‏ that the relationship has already been set before the confirmatory factor analysis, which made the observable variable‏’s specific factor loading‏. By choosing the factor loading that is statistically significant first, with the value higher than 0‏.30^27^ and Cronbach‏’s Alpha ‏≥0‏.70^28^, this could be used in the field as the target indicator for public health‏-related research, which will lower the cost of obesity preventive measures‏.

The assessment of HL for obesity preventive measures in children of ages 9‏–14 showed that most of the subjects had low level of HL ‏(60‏.4‏%), while their obesity preventive behaviors were mostly rated as fair‏. This means that the samples, to some degree, were on the right track and often participated in community health activities‏. One of the factors that shaped up the figures was education as Sirikul‏ found that these kids, at their late time in primary schools in Bangkok, were on the edge of becoming teenagers as girls were more interested in their gender roles and prioritized their physical looks as well as limited the food for the sake of the physical appearance^29^‏. Parents‏’ low educations also lead to overweight children^30^,‏ while parents‏’ nutrition conditions on top of it as children with well‏-educated parents are likely to choose better nutrition path^31^‏. However, children may still have poor diet if parents do not have enough time to look after themespecially nowadays when children are surrounded by the obesogenic environment that accelerates obesity rate with high‏-calorie foods, for example, convenience, advertisement, that encourages children to eat more than their need^32^‏.

The path model analysis between the components of HL for preventive behaviors of obesity showed that the model was consistent with the empirical data‏. The six‏ skills affect the preventive behaviors and that preventive behavior are directly influenced by individual‏’s health knowledge as well as from the other 2 paths which are Path 1 starts from‏ HL that influences managing their health conditions, media, and information literacy and making appropriate health‏-related decision. Additionally, Path 2 starts from accessing the information and services‏ that influenced‏ communicating for added skills, media literacy, and making appropriate health‏-related decision‏. This is consistent concept of HL that skills of receiving and analyzing information will lead to a good HBs^33^‏. Likewise, the relationship between HL and overweight children‏ in China with the sample size of 1305, found that the relativity value between those with low HL and overweight condition were at 0‏.05^34^, HL also is an important indicator of the consumption of healthy food, with the relativity value of‏ 0‏.05^35^‏. Last, the study‏ the eating habits of 7^th^‏-grade students in middle schools in Bangkok were positively related with HL especially decision‏-making skill, with the relativity value of 0‏.05 while the exercising habits were positively related to self‏-management and media literacy at 0‏.01 and 0‏.05, respectively^25^. Therefore, the development of HL in every dimension is required in the development of preventive behaviors of obesity‏.

The analysis of the causal model for obesity preventive measure pointed out that the fundamental intelligence level based on the health knowledge and understanding‏ and accessing the information and services that influences HB‏ for preventing obesity through communicative, interactive, and critical level of HL (effected size were 0‏.76, 0‏.97, and 0‏.55, respectively) corresponded to Nutbeam‏’s model of HL‏ which consisted of 3 levels‏: functional HL, communicative HL, and critical HL^36^‏.

This study has suggestions‏. First, the analysis of the composition of HL that affects the preventive behaviors of obesity has led to HL of children in Thai context, which leads to the assessment of HL in children in order to understand the health situation and sharpen the encouragement of preventive behaviors of obesity‏. Secondly, the assessment should be done on individual and local level for finding the suitable activities for each‏ individual while stimulate and encourage HL accurately for each community‏. Thirdly, further studies need to find other factors that influence HL, which affects the preventive behaviors of obesity, and make the future prediction as strong as possible‏. Lastly,‏ action research needs to be developed and‏ set up a direction for the development of each component of HL that affects the preventive behaviors of obesity, especially the problematic ones as well as those that need an urgent action so that the feasible concept can be manifested‏.

## Conclusions

This developed Health Literacy Scale for Thai childhood overweight can be applied to measure and evaluate the HL level for national policies to improve the health of Thai children‏.

## Acknowledgements

The authors thank the participants include teachers and students of the target schools in this study‏.

## Conflict of interest statement

This work is original and has not been published elsewhere nor is it currently under consideration for publication elsewhere. The authors declare no conflict of interest‏.

## Funding

This study, as a part of research project of Contract Ref‏. No. 3‏/2557, was‏ financially‏ supported by Health Education Division, Department of Health Service Support, and Ministry of Public Health of Thailand‏.

## Highlights

- Health Literacy is the key factor of the obesity preventive behaviors.
- Health Literacy development is improving the cognitive and social skills related health.
- The teachers or providers should use health literacy scale for screening in obesity prevention
